# Are Malaysians Ready to Resume the New Norm? Findings From a Nationwide Study

**DOI:** 10.3389/fpubh.2022.823047

**Published:** 2022-06-03

**Authors:** Sherilyn Pak Cheng Suet, Muhammad Junaid Farrukh, Hee Mei Qi, Zikria Saleem, Muhammad Salman, Aziz ur Rahman, Khang Wen Goh, Long Chiau Ming

**Affiliations:** ^1^Faculty of Pharmaceutical Sciences, UCSI University, Kuala Lumpur, Malaysia; ^2^Faculty of Pharmacy, University of Lahore, Lahore, Pakistan; ^3^Faculty of Data Science and Information Technology, INTI International University, Nilai, Malaysia; ^4^PAP Rashidah Sa'adatul Bolkiah Institute of Health Sciences, Universiti Brunei Darussalam, Gadong, Brunei

**Keywords:** COVID-19, coronavirus, SARS-CoV-2, severe acute respiratory syndrome coronavirus 2, cross-sectional survey

## Abstract

**Objective:**

The study aimed to evaluate the knowledge, attitude, and practices toward the prevention of coronavirus disease 2019 (COVID-19) among the public in Malaysia.

**Methods:**

A cross-sectional study was conducted online among the general public in Malaysia from June 2020 to August 2020 between the second and third wave (Phase 3 of the Recovery Movement Control Order). Participants were conveniently recruited through multiple social media platforms to encourage nationwide participation. A patient-administered questionnaire was used to assess their knowledge, attitude, and practice toward the prevention of COVID-19. For descriptive analysis, percentage, mean, and standard deviation (SD) were used to report demographic characteristics and knowledge, attitude, and practice scores. For inferential analysis, *t*-test, ANOVA, Pearson's correlation, Spearman's correlation, Chi-square test, and Binary Logistic Regression was used to analyze the study variables' differentiation, association, and correlations. The confidence interval selected for this study was 95%.

**Results:**

A total of 420 respondents participated in this survey. The majority of the participants (*n* = 412, 98%) were aware of COVID-19. Most participants (60%) learned about the pandemic through social media. About half of the participants had poor knowledge (45.5%) and a negative attitude (43.3%). Participants residing in urban areas showed good preventive practices as compared to those residing in rural areas (*P* < 0.05). There was a significant association between participants' attitudes and preventive practices toward COVID-19, where the majority of the participants (57.4%) who showed negative attitudes were more likely to follow poor preventive practices.

**Conclusion:**

Despite having good knowledge, participants with a negative attitude toward COVID-19 were less likely to follow the preventive practices of COVID-19. People's mindset and willingness may play an important role to influence their practices. Thus, these are the vulnerable groups, and strategies should be made to change their mindset through proper counseling and education.

## Introduction

An outbreak in the city of Wuhan, by the severe acute respiratory syndrome coronavirus 2 (SARS-Cov 2), also known as coronavirus disease 2019 (COVID-19), has since been classified as a global pandemic (1, 2). This virus has infected many people with low to moderate severity symptoms, including pneumonia, acute respiratory distress syndrome (ARDS), and even multi-organ dysfunction (2).

Malaysia has discovered its first COVID-19 case in January 2020 (3).A total of 4 waves have hit Malaysia from the end of January 2020 to mid-February 2020, followed by the second wave which struck at the end of February to the end of June (4). The third wave, unfortunately, began on the 8th of September and lasted for a few weeks before we were affected by the fourth wave until this day (4, 5). Since then, COVID-19 cases started to spike to more than a thousand during the second wave due to a massive religious event but were still characterized into 3 variants namely type A, B, and C (3). In 2021, the novel coronavirus had mutated into another 3 more forms namely the gamma, delta, and the lambda variant (6).

The Malaysian authorities implemented the first nationwide Movement Control Order (MCO) from March 18 to 31, 2020 to slow the spread of COVID-19 (3). Different lockdown measures were implemented in 2020 and 2021 in different districts/areas based on the number of COVID-19 cases, such as Enhanced Movement Control Order (EMCO), Conditional Movement Control Order (CMCO), Targeted Enhanced Movement Control Order (TEMCO), and Recovery Movement Control Order (RMCO) (7). The Malaysian government, Ministry of Health, front liners, and many others joined forces to flatten the curve and mitigate the outbreak in Malaysia.

Despite the implementation of strict rules and regulations, some citizens of Malaysia did not follow the Standard Operating Procedures (SOPs) of the restricted MCO. They were breaching and flouting the rules and regulations for the sake of business, entertainment, and satisfaction (8, 9). Roadblocks were set up during the MCO to detain unnecessary outings and activities. Unfortunately, 828 citizens were arrested for not complying with the MCO during that period (10). Thus, this explains that a lack of preventive measures was carried out by citizens as it may be due to the lack of knowledge on the threatening virus. With a lack of knowledge, the attitude of Malaysians toward the coronavirus may be the key factor in leading cases.

Unfortunately, there is currently no cure for COVID-19 (11). However, Malaysia received the first batch of Pfizer-BioNTech COVID-19 Vaccine on the 21st of February 2021. Apart from Pfizer-BioNTech, a variety of vaccines namely AstraZeneca, Sinovac, CanSino Biologics, and Sputnik V were brought to Malaysia (12). Although, the vaccines are available but are currently reserved only for front liners (Phase 1) from February to April (13). The citizens of Malaysia have to assist health authorities by strictly following the SOPs and the new norms by wearing a mask, regularly washing and sanitizing hands, and practicing social distancing to prevent and reduce the spread of COVID-19.

Although there are few studies that previously assessed the knowledge, attitude, and practices of COVID-19 prevention, they did not assess these components in detail and did not establish any association between them (14). This research aimed to evaluate the levels of knowledge, attitude, and practices toward COVID-19 and to check the association of these parameters with their preventive practices among Malaysians. It is important that the public correct their mindset on practicing good hygiene and contribute their part to mitigate the virus together with healthcare professionals.

## Materials and Methods

### Study Design

A cross-sectional study design was used to evaluate the knowledge, attitude, and preventive practices of the Malaysian public toward COVID-19. The participants were recruited conveniently from several social media platforms, such as Facebook, WhatsApp, Twitter, and Instagram.

### Sampling Method and Sampling Size

The sampling size was determined based on the current population estimates in Malaysia at 32.73 million in 2020 (11). The sampling size was calculated using Raosoft software by keeping a confidence interval of 95%, a margin of error of 5%, and 50% of the response rate (15). A minimum of 385 respondents was required in this study as shown below. Inclusion criteria consisted of Malaysians above the age of 18 years, consequently, the exclusion criteria consist of non-Malaysians, anyone below 18 as well as the public who are not willing to participate in the survey.


$$\begin{array}{l} n=Z^{2}P(1-P)/d^{2}\\ Z=Z-score\;for\;95\%CI=1.96\\ P(\text{response distribution})=50\%=0.5\\ d(\text{margin of error})=5\%=0.05\\ n={\left(1.96\right)}^{\text{2}}x0.5(0.5)/{\left(0.05\right)}^{\text{2}}\\ n=0.9604/0.0025\\ n=384.16. \end{array}$$

### Study Tools

The questionnaire was adapted from the previous studies. It consisted of 5 sections (14, 16, 17). Section A captured the participant's basic socio-demographic data, including age, sex, religion, marital status, level of education, residential area, monthly income, number of comorbidities, and number of medications. Section B consisted of items to evaluate the knowledge about the coronavirus, and Section C and D consisted of questions regarding attitude and practices toward prevention of COVID-19, respectively. The English version of the questionnaire was translated and back-translated into Malay (Bahasa Malaysia/Melayu) and Mandarin (Bahasa Cina) languages. The translated version of the questionnaire was then reviewed by 2 bilingual academicians and 1 practicing pharmacist.

### Validation of Questionnaire and Pilot Study

For face and content validation, the questionnaire was reviewed by a panel of experts comprising researchers, physicians, academicians, and pharmacists. These experts reviewed the questionnaire based on content relevance, clarity, simplicity, and ambiguity. After revising the questionnaire in light of comments of the expert panel, a pilot study was carried out among 30 subjects to ensure the reliability of the questionnaire formulated (16). The internal consistency was calculated by Cronbach's alpha coefficient, which was 0.606 for knowledge, 0.797 for attitude, and 0.880 for practice.

### Data Collection

Data was collected from the participants through online platforms. Informed consent was provided along with the survey form. Before the survey begins, participants were required to fill in their initials as their signature to indicate they agreed to participate in the study and the information given was accurate to the best of their knowledge. All participants were informed about the purpose of the study.

### Data Analysis

For the 13 knowledge items, each item had 2 choices including correct and wrong answers. The correct answer was given a score of 1, while the incorrect answer was scored 0, with a maximum total score of 13. The attitude scores ranged from 0 to 37 whereas, the total possible score for practices ranged from 0 to 48. The mean and SD of the total scores of knowledge (10.35 ± 1.897), attitude (28.47 ± 6.323), and practices (38.07 ± 7.496) were used to determine the midpoint. Scores higher than the midpoint were classified as “good knowledge,” “positive attitude,” and “good practice” (18, 19).

The obtained data were analyzed using descriptive and inferential analysis where percentage, mean, and SD were used to report demographic characteristics, knowledge, attitude, and practice scores. Whereas, *t*-test, ANOVA, Pearson's correlation, and Chi-square test were used to analyze the study variables' differentiation, association, and correlations. Variables with a *p*-value < 0.1 in the univariate analysis were selected to be modeled using Binary Logistic Regression with the Backward-stepwise method. In the final model only variables with *P* < 0.05 were considered to have a significant influence on practice toward prevention of COVID-19.

## Results

A total of 420 participants took part in this survey. The mean age in this study was 34 years (SD = 15.57, range = 18–95), with a predominance of males (52.9%). The majority of the participants (79.8%) were from a non-medical background. Since COVID-19 has been classified as the world's pandemic, 98.09% (*n* = 412 out of 420) of people who participated in this study had heard about the outbreak with a high percentage of information obtained from social media, such as Facebook, Twitter, and Instagram (60%).

The mean ± SD of Knowledge, Attitude and Practices scores were 10.35 ± 1.897 (79.63%), 28.47 ± 6.323 (76.95%), and 38.07 ± 7.496 (79.31%), respectively. In this study, 45.5% individuals (*n* = 191) had poor knowledge, 43.3% (*n* = 182) had negative attitude, and 42.6% (*n* = 179) had poor preventive practices related to COVID-19.

The details of overall knowledge, attitude, and practice (KAP) scores and participants' sociodemographic characteristics are shown in Table 1. There was a significant difference in mean knowledge scores between age groups, religion, marital status, and race. *Post hoc* analysis revealed that this difference was larger between the 18–29 and 30–49 age categories. Mean attitude scores showed a significant difference between the education levels, where *post hoc* analysis confirmed that this difference was larger between university and secondary school education levels.

**Table 1:** Scores between-demographic difference in knowledge, attitudes and practices.

**Variable**	**Subgroup**	**Mean score** **±SD**		
		**Knowledge**	**Attitude**	**Practices**
Age	18–29	9.98 ± 1.76	29.05 ± 6.14	37.59 ± 7.76
	30–49	10.79 ± 1.72	29.09 ± 6.27	38.33 ± 7.54
	≥50	10.68 ± 2.21	26.53 ± 6.45	38.85 ± 6.81
	*p*-value	<0.05^a^^*^	<0.05^a^^*^	0.358^a^
		0.111^b^	−0.169^b^	0.051^b^
		*r =* 0.023	*r =* 0.001	*r =* 0.295
Sex	Male	10.32 ± 1.80	28.21 ± 6.39	38.13 ± 7.34
	Female	10.39 ± 2.01	28.77 ± 6.25	38.01 ± 7.68
	*p*-value	0.692^d^	0.361^d^	0.864^d^
		0.019^b^	0.045^b^	−0.008^b^
		*r =* 0.692	*r =* 0.361	*r =* 0.864
Race	Chinese	10.27 ± 1.88	28.36 ± 6.31	37.91 ± 7.02
	Malay	10.71 ± 1.52	29.24 ± 6.23	37.71 ± 7.10
	Indian	10.86 ± 1.65	28.27 ± 6.35	39.51 ± 9.49
	Others	8.5 ± 3.13	28.14 ± 7.27	37.86 ± 10.49
	*p*-value	<0.05^a^^*^	0.779^a^	0.539^a^
		−0.005^b^	0.006^b^	0.044^b^
		*r =* 0.924	*r =* 0.901	*r =* 0.368
Religion	Buddhist	10.07 ± 2.03	28.24 ± 6.35	37.40 ± 7.31
	Christian	10.70 ± 1.52	28.37 ± 6.00	39.47 ± 6.20
	Islam	10.49 ± 2.04	28.91 ± 6.63	37.29 ± 8.02
	Hindu	10.83 ± 1.65	27.78 ± 6.66	39.23 ± 10.42
	Others	10.16 ± 1.74	31.47 ± 5.42	38.63 ± 5.18
	*p* value	<0.05^a^^*^	0.251^a^	0.156^a^
		0.098^b^	0.060^b^	0.056^b^
		*r =* 0.044	*r =* 0.219	*r =* 0.253
Marital Status	Single	10.07 ± 1.87	29.06 ± 6.13	37.91 ± 7.44
	Married	10.81 ± 1.86	27.53 ± 6.61	38.39 ± 7.54
	Divorced	10.14 ± 1.86	28.71 ± 4.23	36.57 ± 9.31
	*p*-value	<0.05^a^^*^	0.056^a^^*^	0.713^a^
		0.166^b^	−0.106^b^	0.018^b^
		*r =* 0.001	*r =* 0.031	*r =* 0.714
Highest Level of Education	Secondary School	9.76 ± 2.54	26.51 ± 5.97	35.02 ± 9.33
	College	10.50 ± 1.64	28.76 ± 5.52	39.05 ± 6.24
	University	10.43 ± 1.80	28.81 ± 6.61	38.40 ± 7.32
	*p*-value	<0.05^a^^*^	<0.05^a^^*^	<0.05^a^^*^
		0.053^c^	0.121^c^	0.073^c^
		*r =* 0.275	*r =* 0.013	*r =* 0.135
Relations to studies/ occupation	Health-care related	10.80 ± 1.23	28.36 ± 6.62	37.63 ± 7.69
	Not health-care	10.24 ± 2.02	28.50 ± 6.23	39.80 ± 6.43
	*p*-value	<0.05^a^^*^	0.859^d^	<0.05^d^^*^
		0.120^b^	−0.009^b^	0.116^b^
		*r =* 0.014	*r =* 0.859	*r =* 0.017
Monthly income	<1,000	10.05 ± 2.05	29.22 ± 6.09	38.22 ± 7.04
	1,000–3000	9.85 ± 1.87	27.61 ± 6.65	36.35 ± 8.66
	3,000–5,000	11.13 ± 1.32	28.03 ± 6.54	38.12 ± 7.49
	>5,000	10.91 ± 1.64	28.13 ± 6.24	39.44 ± 6.93
	*p*-value	<0.05^a^^*^	0.191^a^	0.05^a^^*^
		0.215^b^	−0.071^b^	0.060^b^
		*r =* <0.‘05	*r =* 0.148	*r =* 0.218
Residential area	Rural	9.74 ± 1.80	27.46 ± 6.52	36.88 ± 8.84
	Urban	10.62 ± 1.89	28.92 ± 6.19	38.60 ± 6.77
	*p* value	0.353^d^	< 0.05^d^^*^	< 0.05^d^^*^
		0.213^b^	0.107^b^	0.106^b^
		*r =* <0.05	*r =* 0.028	*r =* 0.029

* *P < 0.05 is significant*.

a *ANOVA,*

b *Pearson's correlation,*

c *Spearman's correlation,*

d *Independent t-test, r, Pearson's/Spearman's P-value*.

Participants' response to knowledge statements is summarized in Table 2. Around 96% (*n* = 403) of participants in this study were aware COVID-19 originated from Wuhan, 92.9% (*n* = 390) of participants were aware that a virus was the cause of the disease, however, 7.1% (*n* = 30) of respondents reported that COVID-19 was caused either by bacteria, parasite, or fungi. Around 40% (*n* = 167) of people reported that COVID-19 is a water-borne disease, whereas 60.2% (*n* = 253) gave correct responses.

**Table 2:** Response of study participants for COVID-19 knowledge questions.

**Questions**	**No. of participants (%)**	
	**Correct**	**Incorrect**
Where was the first COVID-19 case recorded?	403 (96.0)	17 (4.0)
Which of following can cause COVID-19?	390 (92.9)	30 (7.1)
Is COVID-19 a water borne disease (spread by water)?	253 (60.2)	167 (39.8)
Coronavirus is transmitted by close contact with infected person.	408 (97.1)	12 (2.9)
Fever, cough and shortness of breath are the most common symptoms of COVID-19.	411 (97.9)	9 (2.1)
Muscle ache, sore throat, and diarrhea could also be the symptoms of COVID-19.	282 (67.1)	138 (32.9)
Patients with underlying chronic diseases (hypertension, diabetes, cardiovascular disease, cerebrovascular disease, chronic respiratory disorders etc.) are at a higher risk of COVID-19.	361 (86.0)	59 (14.0)
Children have less risk of COVID-19 as compared to elderly people.	278 (66.2)	142 (33.8)
Washing hands with soap and water can help in prevention of COVID-19 transmission?	393 (93.6)	27 (6.4)
Wearing face mask is an effective prevention strategy for COVID-19.	405 (96.4)	15 (3.6)
N-95 masks are safer than surgical masks in preventing Covid-19 human to human transmission.	277 (66.0)	143 (34.0)
At this moment, is there any cure for COVID-19?	292 (69.5)	128 (30.5)
Currently, is there an approved vaccine for COVID-19?	330 (78.6)	90 (21.4)

Nearly 77% (*n* = 322) of participants showed a positive attitude toward the statement “COVID-19 can be treated at home without staying in touch with the doctor.” Besides that, 35.2% (*n* = 148) of people were not keen to be vaccinated even if the COVID-19 vaccine was made available. Coughing and sneezing etiquette, such as covering your mouth with a tissue when you sneeze or cough, was essential according to 78.3% (*n* = 329) of Malaysians, whereas 21.7% (*n* = 91) of citizens thought it is not necessary to follow these coughing and sneezing etiquettes. Responses of study participants to COVID-19 attitude questions are shown in Table 3.

**Table 3:** Response of study participants to COVID-19 attitude questions.

**Question**	**No. of participants (%)**	
	**Positive**	**Negative**
Social distancing is unnecessary as long as you are wearing a facemask.	384 (91.5)	36 (8.5)
It is not important to find out information regarding COVID-19 as long as I take care of myself.	376 (89.6)	44 (10.5)
Coronavirus is not a life-threatening infection.	375 (89.3)	45 (10.8)
Coronavirus infection can be treated at home without staying in touch with the doctor.	322 (76.7)	98 (23.3)
If a coronavirus vaccine is available, would you have it?	272 (64.8)	148 (35.2)
It is important to use face mask as a preventive measure.	325 (77.4)	95 (22.6)
It is important to follow cough or sneezing etiquettes. (e.g.,: covering your mouth with tissue when you sneeze or cough).	329 (78.3)	91 (21.7)
Health education has nothing to do with the Coronavirus disease prevention.	349 (83.0)	71 (17.0)
Handling Coronavirus infected patient does not put you at risk of catching COVID-19.	371 (88.3)	49 (11.7)

The COVID-19 preventive practices of the study participants are shown in Table 4. Around 82% (*n* = 343) of respondents cough and sneeze in their upper sleeves when there is no tissue available. Besides that, many have not adapted some good practices like avoiding touching the face, nose, or mouth when their hands are contaminated. Nearly 73% (*n* = 308) of people have shown good practice in this case but 26.6% (*n* = 112) have yet to carry out good practice.

**Table 4:** Response of study participants for COVID-19 preventive practice questions.

**Question**	**No. of participants (%)**	
	**Good**	**Poor**
I follow the advices given by health care professionals on the prevention of COVID-19.	361 (86.0)	59 (14.0)
During the COVID-19 pandemic, I will wear a face mask when I am sick.	373 (88.8)	47 (11.2)
I maintain at least 1-meter distance between myself and others, especially in public.	328 (78.1)	92 (21.9)
I sanitize and wash my hands no matter where I am.	334 (79.5)	86 (20.5)
I cover my nose and mouth with a tissue during sneezing or coughing.	364 (86.7)	56 (13.3)
I throw the used tissue in the trash immediately.	348 (82.8)	72 (17.2)
If no tissue available, I cough or sneeze into my upper sleeve.	77 (18.3)	343 (81.7)
I avoid touching my face (eyes, nose or mouth) with contaminated (dirty) hands.	308 (73.4)	112 (26.6)
I use soap and water to wash my hands quickly after coughing or sneezing or touching contaminated objects like tissue.	326 (77.6)	94 (22.4)
I wear a face mask in the crowds nowadays.	382 (90.9)	38 (9.1)
I comply (obey) to the rules set by the government during the Movement Control order (MCO) period such as staying at home, social distancing, etc.?	370 (88.1)	50 (11.9)
I keep myself updated on the latest information about COVID-19.	339 (80.8)	81 (19.2)

There was no association found between knowledge and preventive practices. However, interestingly a significant association (*P* < 0.01) between participants' attitudes and preventive practices toward COVID-19 was found, where 57.1% (*n* = 104) of the participants who showed negative attitudes were more likely to have poor preventive practices. Univariate analysis was applied to study the relationship between the attitude statements and preventive practices by using the Chi-square test. Variables with *P*-value of < 0.1 in the univariate analysis were selected to be modeled using Binary Logistic Regression (BLR) with the Backward-stepwise method. In the final model, only variables with *P* < 0.05 were considered to have a significant influence on practice toward prevention of COVID-19 as shown in Table 5.

**Table 5:** Binary logistic regression on preventive practices.

**Preventive practices Item**	***B***	**S.E.**	**Wald**	**d*f***	**Sig.**	**Exp(*B*)**	**95% C.I. for EXP(** ***B*** **)**	
							**Lower**	**Upper**
Health education has nothing to do with the Coronavirus disease prevention.	0.098	0.309	0.1	1	0.752	1.103	0.602	2.022
Coronavirus is not a life-threatening infection.	0.164	0.374	0.193	1	0.66	1.179	0.566	2.453
Social distancing is unnecessary as long as you are wearing a facemask.	0.39	0.434	0.808	1	0.369	1.477	0.632	3.459
It is not important to find out information regarding COVID-19 as long as I take care of myself.	0.774	0.359	4.654	1	0.031^*^	2.169	1.073	4.382
Coronavirus infection can be treated at home without staying in touch with the doctor.	0.701	0.251	7.785	1	0.005^*^	2.016	1.232	3.298
If a coronavirus vaccine is available, would you have it?	0.554	0.212	6.865	1	0.009^*^	1.741	1.15	2.635

* *P < 0.05 is significant*.

## Discussion

The overall findings of this study showed that Malaysians have an overall admissible knowledge, positive attitude, and effective preventive measures toward COVID-19. The knowledge of people in Malaysia was significantly associated with age, race, religion, marital status, education level, and monthly income (*P* < 0.05). Based on findings, a total of 54.50% of people had good knowledge, especially during this RMCO. Similar results were seen in a study done in Pakistan, where the majority of the participants showed good knowledge (50.2%) (17).

The study showed that the age group of 30–49 had the highest level of knowledge, followed by adults above 50. This was probably due to the different knowledge acquired from different majors, as well as school types (20). These higher knowledge scores can be possibly due to higher risk perception of contraction and complications from the disease (14). Hence, explaining the pattern for higher knowledge between the age group of 30–49. Other than that, the variation in cultures, norms, and sample size may be different when it is carried out in different studies from different countries (21). These studies were adapted from China, where citizens were brought up with the norm of a communist country, culture, and population enlightening the results of this study (20–22). The lower knowledge of COVID-19 among the 18–29-year-old category can be related to the source of information. Mostly, the youngsters are tech-savvy and rely on information from the internet, which might be misleading. This is consistent with another study, where the age group 18–29 years old category showed the least knowledge of COVID-19 (14). There is a significant association between knowledge and marital status in this study, which might be due to the knowledge shared among married couples, which helps each other improve their knowledge significantly (23). A study found that in Vietnam, married couples tend to have higher knowledge and good preventive practices toward controlling COVID-19 (23).

Indian race reported having higher knowledge compared to other races, whereas race classified as “others” consisted of the underprivileged population (aborigines/orang asli), which reported the lowest knowledge. It could be due to a lack of internet and social media access for these people. The study expects to provide information to policymakers for policy improvement. Healthcare professionals have a significant role to play in educating policymakers on the need to include socially disadvantaged populations and the merit of universal approaches to COVID-19 prevention.

This study showed a lack of association between knowledge and the education level of the participants as the level of knowledge in college was higher than at university levels. Thus, the findings were not identical to a study from Saudi Arabia, where participants with a higher level of education had a higher level of knowledge (23). According to a study in Indonesia, most students were aware of the existence of COVID-19, but only a few had good knowledge of the virus (24). The news on the existence of the virus may be the topic of many during daily conversations but deep knowledge might not be discussed during the conversations leading to awareness of the existences of COVID-19, but lesser knowledge based on this Indonesian study (23). Some Malaysians may also have a higher level of education but might tend to overlook the importance of being updated on news or related articles regarding COVID-19 due to their busy schedule.

Based on this study, only 60.2% of people were aware that COVID-19 is not a waterborne disease, thus, making as much as 39.8% of the public, who are unaware of this information. In contrast, another study done in Malaysia showed a lower percentage of people (43.3%), who believed that COVID-19 is not airborne (14). Most Malaysians were aware only of the most common symptoms, such as fever, dry cough, and fatigue. However, the other studies conducted in Malaysia reported that 35% of Malaysians were unsure of these symptoms of COVID-19, thus, supporting the evidence of this study's results (14).

Most participants learned about the pandemic through social media. The information obtained from online sources might not be reliable. Most of them tend to read the news without checking the credible source, such as health authorities or the government, and then afterward, share the information with their family and friends. This might create confusion among the public by giving out incorrect information through a lot of fake news and false accusations despite the information given by the authorities (25, 26). This might affect a person's knowledge level as they acquire information online, describing the reason behind those with lower knowledge of COVID-19.

More than three quarters of the participants had a positive attitude toward COVID-19. In this research, the participant's attitude is corresponding to age, marital status, education level, and residential area. This study also showed that participants had a lower level of positive attitude compared to a study done in Pakistan (65.4% with a positive attitude) and China (73.81% with a positive attitude) (13, 20). This might be due to the different mindsets and mentality of Malaysians coming from different cultures and races where they adapt to learning differently (27). Some may adopt the teachings of one's parents and one may adapt them through reading newsletters, online sources, or even listening to the radio and television (27).

The majority of the participants agreed that it is important to practice social distancing, especially in crowded areas, to prevent the spread of the virus. One-meter distancing decreases the chance of virus transmission from the infected persons and contaminated surfaces (28). Similar findings were reported in another study (29). A study mentioned that with the right and optimistic attitude, one would automatically carry out positive practices supporting the relationship and association of attitude and preventive practices in this study (30).

This study has shown that there was no association between participants' knowledge and their attitude. This indicates that although one may have good knowledge, they may not have a positive mindset and attitude toward COVID-19. Different findings were found in a study from China, they reported that people with higher knowledge have a better or positive attitude toward the disease (30). The possible consequences of the public with good knowledge but negative attitude is associated with poor compliance in following the instructions given by health authorities and might cause further spread of COVID-19.

As per this study, nearly half of the candidates showed good preventive measures (practices) toward the disease. On top of that, prevention practices for COVID-19 were correlated with education level, occupation, and residential area. This study reported that Malaysians' attitude is in line with the practices they carry out in their daily routines. This also means that people with a more positive attitude would be more prone to carrying out a better preventive practices. Based on findings, practices are affiliated with the correlation of studies or occupations toward healthcare settings and residential areas. The percentage of good preventive practices in this study (57.40%) was higher than a study in Pakistan (36.5%) (17).

The study also reported that most of the participants take the necessary precautions such as following advice given by the health care professionals, wearing a face mask whenever sick, keeping a good hand hygiene, maintaining a one-meter distance especially in crowded areas, and so on. The findings were supported by a Malaysian study that stated that Malaysians strictly practice social distancing to improve the situation and prevent the spread of the virus, especially during the pandemic (29). Misinformation that is often found on social media has also influenced some of the public into doing the wrong practices, as well as causing mental breakdowns according to a new study done in Korea (31). It was also reported that the pandemic also caused a large sum of papers and articles that were not properly reviewed to be published, thus, increasing the misinformation circulation among the public.

An interesting finding was also established regarding wearing of facemasks in this new norm. Many participants reported that they wore a facemask in crowded areas, especially when they are sick. Consequently, many participants also stated that they often sanitize their hands no matter where they are. The local media reported that it increased the demand for hand sanitizers and face masks among Malaysians (32). This explained that Malaysians fear the virus and disease that they currently face, to the point that stores were short of stocks. It was believed that the public was buying to stock up because many other countries that supply these essential items were also facing shortages (25–34). The cause of panic buying and massive stock up could be the fear and confusion that the public faced during the pandemic. Some studies also mentioned that awareness must be brought to the public to allow them to understand the situation and stop the fear (17, 35, 36). Education from health authorities should be provided on multiple platforms to help the public to understand the situation better.

Finally, there was no association found between knowledge and preventive practices. The reason might be because a person with good knowledge may not want to perform the said preventive practices toward COVID-19. There may be various reasons which might include a different behavior and mindset would relate to the consequences of their preventive practices. As found in this study, one could be aware that sneezing into the upper sleeve is not a good practice but carrying out these practices anyway for the sake of convenience. This explains that even by understanding the consequences of sneezing into the upper sleeve, preventive practices may not be done by the person because it may be more convenient to perform bad practice rather than good practice.

Ultimately, this study had indicated a significant association between participants' attitudes and preventive practices toward COVID-19 according to the univariate analysis and binary logistic regression. In the end, the results showed that there were 3 most significant attitudes associated with poor preventive practices, namely, having the mentality that it is not important to find out information on COVID-19 as long as each of them take care of themselves' “COVID-19 can be treated at home without staying in touch with the doctor,” and “not very keen on having a vaccine if it is available in Malaysia”. Some of these thoughts were affecting their practices toward COVID-19 by showing that the more negative a person's attitude is, the more they are to carry out poorer practices in this study. Thus, this study tells us that it is important to provide education *via* multiple platforms to instill the mindset and mentality of the people in Malaysia.

## Limitations

This research was conducted online *via* multiple social media platforms, there is a possibility of biasness toward some underprivileged populations (aborigines/orang asli) that may not have access to social media. A higher percentage of samples came from the Chinese race. Another limitation found in this study is that the respondents may answer questions based on what they think is right instead of what they have been practicing in their daily lives. Data was collected by convenient sampling, which is non-probabilistic nature of the sampling strategy, which is vulnerable to selection bias.

## Conclusion

In a nutshell, this study uncovered that despite having good knowledge, participants with a negative attitude toward COVID-19 were less likely to follow the preventive practices of COVID-19. People's mindset and willingness may play an important role to influence their practices. Thus, these are the vulnerable groups, and strategies should be made to change their mindset through proper counseling and education. Healthcare professionals should educate the public in a way to change their mindset and make them act responsibly.

## Data Availability Statement

The original contributions presented in the study are included in the article/Supplementary Material, further inquiries can be directed to the corresponding author/s.

## Ethics Statement

Ethical approval was obtained from the UCSI university Ethics Committee (Ref. no. IEC-2020-FPS-043). Written informed consent was obtained from all the participants before they participated in the study.

## Author Contributions

MF designed the study. Data collection was conducted by SS, MF, HQ, and ZS. Data were evaluated by MF, MS, and AR. SS, MF, HQ, and ZS wrote the first draft of the manuscript. LM and KG assisted in data evaluation and reviewed the manuscript. All authors read and approved the final manuscript. All authors contributions to this work comply with Frontiers in Public Health authorship recommendations.

## Conflict of Interest

The authors declare that the research was conducted in the absence of any commercial or financial relationships that could be construed as a potential conflict of interest.

## Publisher's Note

All claims expressed in this article are solely those of the authors and do not necessarily represent those of their affiliated organizations, or those of the publisher, the editors and the reviewers. Any product that may be evaluated in this article, or claim that may be made by its manufacturer, is not guaranteed or endorsed by the publisher.

## Abbreviations

SARS-Cov 2: Severe Acute Respiratory Syndrome Coronavirus 2
COVID-19: Coronavirus Disease 2019
ARDS: Acute respiratory Distress Syndrome
MCO: Movement Control Order
EMCO: Enhanced Movement Control Order
CMCO: Conditional Movement Control Order
TEMCO: Targeted Enhanced Movement Control Order
RMCO: Recovery Movement Control Order
SOPs: Standard Operating Procedures
KAP: Knowledge, Attitude and Practice
BLR: Binary Logistic Regression.
