# Supplementary material for: Are Malaysians Ready to Resume the New Norm? Findings From a Nationwide Study
Source: Front Public Health. 2022 Jun 3;10:823047. doi: 10.3389/fpubh.2022.823047 (PMC9205470; doi:10.3389/fpubh.2022.823047)
Supplement: Supplementary file 1 [file Data_Sheet_1.pdf]

Evaluation of Knowledge, Attitudes, Practices towards prevention of Coronavirus (COVID-19) infection among general public in Malaysia

SECTION A: BACKGROUND INFORMATIONON OF RESPONDENT

1.	**Age**	________________________ years
2.	**Gender**	☐ Male ☐ Female
3.	**Race**	☐ Chinese ☐ Malay<br>☐ Indian ☐ Other (please state) ________________
4.	**Religion**	☐ Buddhist ☐ Christian<br>☐ Islam ☐ Hindu<br>☐ Other (please state) ________________
5.	**Marital status**	☐ Single ☐ Married ☐ Other (please state) __________________
6.	**Highest Level of Education**	☐ Secondary school<br>☐ College<br>☐ University<br>☐ Other (please state) __________________
7.	**Employment status**	☐ Employed<br>☐ Self-employed<br>☐ Unemployed<br>☐ Student<br>☐ Housewife<br>☐ Other (please state) __________________
8.	**Relations to studies/ occupation**	☐ Health-care related ☐ Not health-care related

9.	**Monthly income**	☐ <1000 ☐ 1000-3000 ☐ 3000-5000 ☐ >5000
10.	**Residential area**	☐ Rural (Outskirt) ☐ Urban (City)
11.	**Regions**	☐ Central (Western)<br>☐ Northern<br>☐ Southern<br>☐ Eastern<br>☐ Sabah/ Sarawak
12.	**Do you have any health problems ?**	☐ No<br>☐ Yes (if YES, please specify) ___________<br>☐ Not sure
13.	**Are you from Klang Valley?**	☐ Yes ☐ No
14.	**Have you heard about the recent Coronavirus disease outbreak (COVID-19)?**	☐ Yes ☐ No ☐ Not sure

15.	**If yes, where did you first learn about this Coronavirus outbreak (COVID-19)?**	☐ Friends/family/relatives/neighbors<br>☐ Social media (Facebook/twitter/Instagram)<br>☐ Television/radio<br>☐ Newspapers<br>☐ University/College<br>☐ Healthcare worker<br>☐ Others (Please specify) ______________

SECTION B: Knowledge of COVID-19

1.	**Where was the first COVID-19 case recorded?**<br>☐ Wuhan<br>☐ Beijing<br>☐ Shanghai<br>☐ Xi'an<br>☐ Don't know
2.	**Which of following can cause COVID-19?**<br>☐ Fungi<br>☐ Bacteria<br>☐ Virus<br>☐ Parasite<br>☐ Don't know
3.	**Is COVID-19 a water borne disease (spread by water)?**<br>☐ Yes ☐ No ☐ Not sure

4.	**Coronavirus is transmitted by close contact with infected person.**<br>☐ Yes ☐ No ☐ Not sure
5.	**Fever, cough and shortness of breath are the most common symptoms of COVID-19.**<br>☐ Yes ☐ No ☐ Not sure
6.	**Muscle ache, sore throat, and diarrhea could also be the symptoms of COVID-19.**<br>☐ Yes ☐ No ☐ Not sure
7.	**Patients with underlying chronic diseases (hypertension, diabetes, cardiovascular disease, cerebrovascular disease, chronic respiratory disorders etc.) are at a higher risk of COVID-19.**<br>☐ Yes ☐ No ☐ Not sure
8.	**Children have less risk of COVID-19 as compared to elderly people.**<br>☐ True ☐ False ☐ Not sure
9.	**Washing hands with soap and water can help in prevention of COVID-19 transmission?**<br>☐ Yes ☐ No ☐ Not sure
10.	**Wearing face mask is an effective prevention strategy for COVID-19.**<br>☐ Yes ☐ No ☐ Not sure

11.	**N-95 masks are safer than surgical masks in preventing Covid-19 human to human transmission.**<br>☐ Yes ☐ No ☐ Not sure
12.	**At this moment, is there any cure for COVID-19?**<br>☐ Yes ☐ No ☐ Not sure
13.	**Currently, is there an approved vaccine for COVID-19?**<br>☐ Yes ☐ No ☐ Not sure

SECTION C: ATTITUDES TOWARDS COVID-19

1.	**Social distancing is unnecessary as long as you are wearing a facemask.**<br>☐ Strongly agree<br>☐ Agree<br>☐ Neither agree nor disagree<br>☐ Disagree<br>☐ Strongly disagree
2.	**It is not important to find out information regarding COVID-19 as long as I take care of myself.**<br>☐ Strongly agree<br>☐ Agree<br>☐ Neither agree nor disagree<br>☐ Disagree<br>☐ Strongly disagree

3. **Coronavirus is not a life-threatening infection.**

☐	Strongly agree
☐	Agree
☐	Neither agree nor disagree
☐	Disagree
☐	Strongly disagree

4. **Coronavirus infection can be treated at home without staying in touch with the doctor.**

☐	Strongly agree
☐	Agree
☐	Neither agree nor disagree
☐	Disagree
☐	Strongly disagree

5. **If a coronavirus vaccine is available, would you have it?**

☐	Strongly agree
☐	Agree
☐	Neither agree nor disagree
☐	Disagree
☐	Strongly disagree

6. **It is important to use face mask as a preventive measure.**

☐	Strongly agree
☐	Agree
☐	Neither agree nor disagree
☐	Disagree
☐	Strongly disagree

7. **It is important to follow cough or sneezing etiquettes. (E.g.: covering your mouth with tissue when you sneeze or cough).**

☐	Strongly agree
☐	Agree
☐	Neither agree nor disagree
☐	Disagree
☐	Strongly disagree

8. **Health education has nothing to do with the Coronavirus disease prevention.**

☐	Strongly agree
☐	Agree
☐	Neither agree nor disagree
☐	Disagree
☐	Strongly disagree

9. **Handling Coronavirus infected patient does not put you at risk of catching COVID-19.**

☐	Strongly agree
☐	Agree
☐	Neither agree nor disagree
☐	Disagree
☐	Strongly disagree

SECTION D: PRACTICES RELATED TO COVID-19

1. **I follow the advices given by health care professionals on the prevention of COVID-19.**

☐	Never
☐	Rarely
☐	Sometimes
☐	Most of the times
☐	Always

2. **During the COVID-19 pandemic, I will wear a face mask when I am sick.**

☐	Never
☐	Rarely
☐	Sometimes
☐	Most of the times
☐	Always

3. **I maintain at least 1-meter distance between myself and others, especially in public.**

☐	Never
☐	Rarely
☐	Sometimes
☐	Most of the times
☐	Always

4.	**I sanitize and wash my hands no matter where I am.**<br>☐ Never<br>☐ Rarely<br>☐ Sometimes<br>☐ Most of the times<br>☐ Always
5.	**I cover my nose and mouth with a tissue during sneezing or coughing.**<br>☐ Never<br>☐ Rarely<br>☐ Sometimes<br>☐ Most of the times<br>☐ Always
6.	**I throw the used tissue in the trash immediately.**<br>☐ Never<br>☐ Rarely<br>☐ Sometimes<br>☐ Most of the times<br>☐ Always
7.	**If no tissue available, I cough or sneeze into my upper sleeve.**<br>☐ Never<br>☐ Rarely<br>☐ Sometimes<br>☐ Most of the times<br>☐ Always

8.	**I avoid touching my face (eyes, nose or mouth) with contaminated (dirty) hands.**<br>☐ Never<br>☐ Rarely<br>☐ Sometimes<br>☐ Most of the times<br>☐ Always
9.	**I use soap and water to wash my hands quickly after coughing or sneezing or touching contaminated objects like a tissue.**<br>☐ Never<br>☐ Rarely<br>☐ Sometimes<br>☐ Most of the times<br>☐ Always
10.	**I wear a face mask in the crowds nowadays.**<br>☐ Never<br>☐ Rarely<br>☐ Sometimes<br>☐ Most of the times<br>☐ Always
11.	**I comply (obey) to the rules set by the government during the Movement Control order (MCO) period such as staying at home, social distancing, etc.?**<br>☐ Never<br>☐ Rarely<br>☐ Sometimes<br>☐ Most of the times<br>☐ Always

12.	**I keep myself updated on the latest information about COVID-19.**

☐	Never
☐	Rarely
☐	Sometimes
☐	Most of the times
☐	Always
